# Factors that may Facilitate or Hinder a Family-Focus in the Treatment of Parents with a Mental Illness

**DOI:** 10.1007/s10826-013-9895-y

**Published:** 2014-03-15

**Authors:** Camilla Lauritzen, Charlotte Reedtz, Karin Van Doesum, Monica Martinussen

**Affiliations:** 1 Center for Child and Youth Mental Health and Welfare, Uit—Arctic University of Norway, Tromsø, Norway; 2 Behavioural Science Institute, Mental Health Centre, Radboud University Nijmegen and Mindfit, Deventer, The Netherlands

**Keywords:** Family focus, Child focus, Parenting, Child mental health prevention, Implementation, Parental mental health

## Abstract

Children with mentally ill parents are at risk of developing mental health problems themselves. To enhance early support for these children may prevent mental health problems from being transmitted from one generation to the next. The sample (*N* = 219) included health professionals in a large university hospital, who responded to a web-based survey on the routines of the mental health services, attitudes within the workforce capacity, worker’s knowledge on the impact of parental mental illness on children, knowledge on legislation concerning children of patients, experience, expectations for possible outcomes of change in current clinical practice and demographic variables. A total of 56 % reported that they did not identify whether or not patients had children. There were no significant differences between the groups (identifiers and non-identifiers) except for the two scales measuring aspects of knowledge, i.e., Knowledge Children and Knowledge Legislation where workers who identified children had higher scores. The results also showed that younger workers with a medium level of education scored higher on Positive Attitudes. Furthermore, workers who reported to have more knowledge about children and the impact of mental illness on the parenting role were less concerned about a child-focussed approach interfering with the patient-therapist relation.

## Introduction

Children of parents with a mental illness are at risk for developing mental health-problems themselves (Beardslee et al. 1998; Hosman et al. 2009; Reupert and Maybery 2007; Rutter and Quinton 1984). According to US studies, 58 % of children with serious mental emotional disturbance have a history of family mental illness, and 40 % have a history of parent psychiatric hospitalization (Biebel et al. 2004). Studies have shown that the risk of trans-generational transmission of psychopathology is high (Downey and Coyne 1990; Goodman and Gotlib 1999; Hosman et al. 2009). The transmission mechanism of parental mental illness to offspring is generally thought to be multifactorial (Garley et al. 1997). Little can be done to alter a child’s genetic predisposition. However, many studies have found that transmission of psychiatric risk is mediated by the way parents interact with their children (Hosman et al. 2009). Factors such as dysfunctional family interaction, insensitive responsiveness, low involvement with the child, low monitoring and hostility as well as child maltreatment, may result from parental psychopathology (Bauer and Webster-Stratton 2006; Foster et al. 2008; Gardner et al. 1999; Granic and Patterson 2006). Further, studies have demonstrated that infants of depressed mothers are found to be insecurely attached more often compared to the normal population (Cicchetti et al. 1998).

Treatment of patients who are parents is more likely to be successful if the treatment includes consideration of their parental role. In a study of state mental health data in the USA, mothers with mental illness identified motherhood as a primary factor for treatment, and reported that not being able to parent their children compromised their well-being and impeded their progress toward recovery (Biebel et al. 2004). Interventions providing families with information about depression, the effect of stress and depression on functioning, and ways to recognize and manage stress significantly reduces depressive and anxiety symptoms in both depressed parents and their offspring (Muñoz et al. 2012). Furthermore, there is evidence to suggest that supporting these children may strengthen their mental health (Maybery and Reupert 2009). For these reasons, it is appropriate to include a child perspective in the treatment of mentally ill parents. (Brockington et al. 2011; Hosman et al. 2009; Van Doesum et al. 2005). There are, however, barriers to achieving this.

In Norway, researchers, practitioners and politicians have increasingly focused on children of parents with a mental illness. There is a general awareness that these children are at risk and therefore need support in order to prevent developing mental health problems themselves. A Norwegian study showed that the services available to these children were insufficient (Aamodt and Aamodt 2005). According to this report, most hospitals and institutions for adults have not offered services to children and relatives. Routines to register whether patients are also parents have been random, unpredictable and fragmentary. Subsequently, providing necessary support and assistance for these children has also been unsystematic. However, the results also showed a consensus among professionals in the field that a change in practice is needed, so that these children may be identified and offered adequate support and help. Consequently, Norwegian authorities have made several changes to the existing legislation (the Health Personnel Act) effective from January 2010 (Norwegian Ministry of Health and Care Services 2010). The intention behind the legislative amendments was to increase early identification and to encourage processes that enable children and parents to better deal with the situation when a parent is seriously ill (Norwegian Health Directorate 2010).

Mayberry and Rupert (2009) concluded that there is a large gap between what psychiatric services should provide and what they in fact practice when it comes to implementing a family focus in adult mental health care. Implementing change in mental health care for adults requires a major shift of focus, which involves innovative efforts at the organizational level and attitude change within the workforce capacity (Maybery and Reupert 2006). In previous research, many factors are shown to limit the support for families within the mental health services (Maybery and Reupert 2009; Korhonen et al. 2008a). Important factors involve policy and management in adult mental health care, such as lack of policy and guidelines, and inadequate resource allocation related to such things as provision of time and high client workloads (Maybery and Reupert 2009).

However, other factors include variables characterizing professionals in the workforce. Korhonen et al. (2009) stated that there are many factors limiting the support for families within mental health services, for instance health professionals’ qualifications. Maybery and Reupert (2006) found that mental healthcare workers reported they had limited skills related to working with children, dealing with parenting issues and working with families in general. Although mental healthcare workers acknowledged the importance of a family focused practice, they simultaneously reported knowledge and skill deficits in their practice related to including a child perspective and issues of parenting competence (Korhonen et al. 2008a; Maybery and Reupert 2006).

Furthermore, some studies have found that worker attitudes may be problematic when attempting to integrate a child focus in mental health care for adults. Korhonen et al. (2008b) documented that mental health nurses do not regard the children of patients as their responsibility. Some workers may believe that family members may cause or increase the mental illness by laying extra burdens on the patient (Maybery and Reupert 2009), and therefore hesitate to include a family perspective in their treatment of patients. The professional background of mental health workers may also be related to attitudes. Maybery and Reupert (2009) found that there was a connection between educational background and attitudes related to family-focused work. According to this review psychologists had more positive attitudes towards a more family-focused approach in clinical practice than did psychiatrists and social workers. Korhonen et al. (2008a) found that there was a strong relationship between nurses trained within a family-focused treatment approach and positive attitudes towards incorporating a child perspective in adult mental health care.

Based on existing research, it seems that one key to implementing a more family-focused practice may be to focus on the professionals in the workforce (Barry and Jenkins 2007; Maybery and Reupert 2009; Reupert and Maybery 2008). However, in order to understand why there is a discrepancy between the services delivered in mental health care and the existing knowledge of the importance of a family-focused practice, it is important to study workforce attitudes more extensively. Unless the workforce recognizes or accepts the premise that a change is needed, an innovative project has little hope of surviving (Fixsen et al. 2005). By examining attitudes, we may find important predictors for workforce resistance to identifying and supporting parents and children in mental health services for adults.

## Aims of the Study

The purpose of the present study was to investigate to what extent the workforce in the adult mental health care identifies whether or not patients have children and their attitude towards including a family focus in treatment. A secondary goal was to examine differences between professionals who identify the children of the patient and those who do not in terms of worker attitudes towards a family focused practice, expectations for positive effects of identification on the children and worker knowledge about children and parenting and the new legislation. Lastly, another aim was to study which factors predicted workforce attitudes by examining the following predictors: age, gender, education, knowledge about the impact of parental mental illness on children and parenting, knowledge about legislation concerning children of patients, and expectations for possible outcomes of change in current clinical practice.

## Method

### Participants

Participants in this study are the staff and managers at psychiatric clinics of a large university hospital in Northern Norway that are implementing new routines for identifying children of patients in the age of 0 up to 18 years old. The university hospital runs several decentralized clinics throughout the region. There were 16 outpatient and inpatient clinics participating, serving a large geographical area of 31 municipalities. The total workforce, including 436 participants, was asked to answer a baseline questionnaire prior to the initial process of implementing new routines. The recruitment for participation was done by a written invitation sent out by e-mail. In the participating clinic (and throughout Norway), the workforce is organized in interdisciplinary treatment teams, where all members of the team, regardless of educational background, are responsible for treatment of the patient. The teams consisted of general nurses, psychiatric nurses, psychologists, psychiatrists and special teachers. Many team-members have a sub-bachelor level education, and take part in the teams as occupational therapists or social therapists. Some of them are medicine or psychology students, and others have been trained in apprenticeship schemes at the upper-secondary level to be health personnel.

A total of 219 individuals responded, representing a response rate of 50 %. The respondents were 76 % women, of which the majority was between 30 and 50 years old. The majority of the respondents were women, and this may subsequently have biased the results. We therefore conducted an analysis of the total workforce in terms of gender. Of the total workforce the percentage of men amounted to 29 %, which was a little bit higher than in our sample (24 %).

Detailed demographic information is presented in Table 1.

**Table 1:** Descriptive statistics for demographic variables (*N* = 219)

Gender		
Women	166	76 %
Men	52	24 %
Age (years)		
< 30	25	11 %
31–40	55	26 %
41–50	57	26 %
51–60	60	27 %
>60	21	10 %
Level of education		
High	30	14 %
Medium	92	42 %
Low	82	37 %
Contact with patients		
Yes	213	97 %
No	4	3 %
Management responsibilities		
Yes	40	18 %
No	178	82 %

The study was approved by the data protection supervisor at the University Hospital of Northern Norway, and was conducted in line with the Helsinki Declaration of ethical principles for medical research involving human subjects published by the World Medical Association (World Medical Association 2008).

### Experimental Design

This study is part of a longitudinal (pre-post-1 year follow-up) study monitoring the implementation of new routines in a large university hospital in Northern Norway. The study design has been published as a protocol article (Reedtz et al. 2012). After the new legislation, staff in hospitals around Norway was informed about the new legislation and the reasons for the demand for new clinical practice. Child responsible staff were appointed in all wards in each hospital. In our project we introduced new routines and practice through the implementation of the interventions *Family Assessment Form* and *Child Talks* in the hospital. The family assessment form is an intervention for practitioners to identify and register children of mentally ill parents, and their needs. The intervention *Child Talks* is a health-promoting and preventive intervention where the mental health worker talks with the family about the children’s situation and needs. This intervention was developed and manualized in the Netherlands by van Doesum and Koster (2008). In the present study only baseline data are presented. An electronic survey questionnaire was used, where reminders were sent out at three different times to all non-respondents. The questionnaires were completed anonymously.

### Measures

The questionnaire included the following topics:

#### Demographic and Work Characteristics

Personal demographic variables included age, gender, discipline and education, in addition to single items on work characteristics such as management responsibilities and current position. Based on the length of education related to nine different disciplines, education was divided into three groups: low (below bachelor level), medium (bachelor), and high (master/equivalent or higher).

#### Routines for Identification

One question was included: “Do you identify children of patients?” Possible answers were *yes* or *no*.

#### Description of Knowledge

Materials for assessing knowledge on the status quo in regular practice and changes in clinical practice were based on the *Keeping Families and Children in Mind Online Resource*—*Evaluation, pre*-*training survey*. This measure has been evaluated in Australia, and was reported to be a useful tool in this context (Mayberry et al. 2011). The questionnaire was adapted to the Norwegian context to assess knowledge within regular practice in the organization for dealing with children of mentally ill parents with permission of the authors. Items included questions on level of knowledge about children of mentally ill parents and the new legislation. The total number of items was ten. The items were answered on a five point Likert-scale from “to a very large extent” (5) to “to a very little extent” (1). A sample question was: “To what extent would you say you have knowledge about the consequences of mental illness for the parenting role?” Principal component analysis (varimax rotation) indicated a two-component structure, where one group of items tapped into self-assessed knowledge on legislation and one group of items tapped into self-assessed knowledge on parental mental illness and consequences for children in general. Two scales were computed based on mean scores of the respective items. The computed Cronbach’s Alpha was .73 for the Knowledge Legislation scale and .90 for the Knowledge Children scale.

#### Expectations Regarding Effects of Implementing an Intervention

Items included questions about the expected outcome for patients and children as a result of the intervention, e.g.: “I believe conversations with and about children may contribute to the improvement of the life situation for children of mentally ill parents”. Four items were answered on a five-point Likert-scale from “to a very large extent” (5) to “to a very little extent” (1). The computed Cronbach’s Alpha for Expectations Intervention was 93. The scale was computed as mean scores of the included items.

#### Attitudes Towards Including the Children’s Perspective in Mental Health Care for Adults

The scale included eleven items. The items were rated on a five-point scale, from “to a very large extent” (5) to “to a very little extent” (1), where score five refers to the most positive attitudes. Principal component analysis (varimax rotation) indicated two components. One group of items tapped into concerns regarding the impact on the patient-therapist relation, e.g.: “It may interfere with the patient-therapist relation if we address parenting issues”. The other group of items tapped into attitudes towards including a child perspective in the treatment of mentally ill parents, e.g. “We should offer support to patients who are parents”. Two scales were computed based on the mean scores of the respective items. The alpha for the Concerns Patient-Therapist was .77 and the alpha for the Positive Attitudes towards a child perspective was .93.

### Data Analyses

All statistical analyses were performed with SPSS (Version 17). Principal component analysis (varimax rotation) was used to reduce the number of items on Attitudes and Knowledge to a smaller number of dimensions. Independent sample t-tests were used to test the differences between the workers who said they identify children and those who said they did not. Correlation analyses were performed to explore the relationship between core variables in the study. The prediction of the two attitudes scales was examined using multiple linear regression analyses. The regression analyses included the following independent variables; age, gender, level of education, knowledge (Knowledge Legislation and Knowledge Children), and finally, expectations (Expectations Intervention). The three groups representing different educational levels (high, medium and low) were coded by means of two dummy variables. The reference category was high education and the two dummy variables were coded: D_medium_ = 1 if medium education and 0 otherwise, and D_low_ = 1 if low education and 0 otherwise. Staff with high education mainly refers to medical doctors and clinical psychologists, medium education refers to psychiatric nurses and general nurses, and low education refers to other health personnel and assistants with education lower than Bachelor level. Pairwise missing was used for descriptive analyses and t tests, and listwise missing was used for the regression analyses. In general, the data set had few missing values.

## Results

In general, the respondents had positive attitudes towards the identification of whether or not patients had children as indicated by the high mean values of both identifiers and non-identifiers on positive attitudes (Table 2). However, of the total sample of 219 who reported that they worked directly with patients, 56 % did not register whether patients had children. Descriptive statistics on sample attitudes, expectations and knowledge about children are displayed in Table 2.

**Table 2:** Independent samples t tests of differences between identifiers and non-identifiers in terms of attitudes, knowledge, and expectations

	Workers who *do* identify *N* = 93		Workers who *do* identify *N* = 119		*t*	Cohen’s d
	*M*	*SD*	*M*	*SD*		
Positive attitudes	4.39	0.52	4.47	0.61	−0.97	−0.17
Concerns patient-therapist	2.27	0.72	2.46	0.76	−1.79	−0.27
Knowledge children	3.61	0.57	3.29	0.63	3.91***	0.50
Knowledge legislation	3.13	0.79	2.75	0.64	3.81***	0.42
Expectation intervention	4.27	0.53	4.24	0.66	0.36	0.03

* *p* < .05, ** *p* < .01, *** *p* < .001 (two-tailed test)

Independent sample t-tests were conducted to compare means between the group who did identify children and workers who did not (see Table 2). There were no significant differences between the groups except for the two scales measuring knowledge: Knowledge Children and Knowledge Legislation. Those who reported that they did identify children had more knowledge about children in general compared to those who did not identify the children. The difference in terms of Cohen’s d was 0.50 for Knowledge Children and 0.42 for Knowledge Legislation, which represents medium effects according to Cohen’s criteria (1988).

To examine which variables predicted attitudes, a multiple regression analysis was conducted with the two different attitude scales as dependent variables. The results of the regression analyses are displayed in Table 3. The independent variables were: age, gender, level of education, knowledge of legislation, and knowledge about children and expectations for the intervention. Our first regression analysis included three significant predictors: age, one dummy variable involving education and Expectations Intervention. This model explained a total of 34 % of the variance. The findings indicated more positive attitudes among younger participants with a medium educational level (compared to high level), and with positive expectations for the intervention. The second regression analysis for predicting Concerns Patient-Therapist had three significant predictors, which were Knowledge Children, Education Low, and Expectations Intervention. The higher the reported level of knowledge about children, the less concerned they were about the family focus interfering with the patient-therapist relationship. Those with a lower educational level were more concerned about the family focus interfering with the patient-therapist relationship. Additionally, those who expected that the intervention would benefit children of mentally ill parents were less concerned about a family focus interfering with the patient-therapist relationship. This model explained a total of 14 % of the variance.

**Table 3:** Multiple regression analysis for predicting attitudes towards a child perspective

Variable	Positive attitudes	Concerns patient-therapist
	β	β
Age	−0.23**	−0.03
Gender	−0.06	0.09
Education D_medium_	0.14*	0.04
Education D_low_	0.06	0.14*
Knowledge legislation	−0.02	0.04
Knowledge children	0.09	−0.26**
Expectations intervention	0.47*	−0.16*
*R* ^2^	0.34	0.14
F	14.97***	4.83***

*N* = 212. Gender was coded 0 = female, 1 = male. Educational levels (high, medium and low) were coded by means of two dummy variables. The reference category was high education and the two dummy variables were coded: D_medium_ = 1 if medium education and 0 otherwise, and D_low_ = 1 if low education and 0 otherwise

* *p* < .05, ** *p* < .01, *** *p* < .001

## Discussion

Based on existing research on workforce attitudes relating to children of mentally ill parents (Maybery and Reupert 2009), we expected to find barriers within mental health services for adults involving identification of the children, providing support for the patient in the parenting role, and offering support to the patients’ children. The majority of participants in this study had positive attitudes towards supporting patients in their parenting role and supporting their children. However, less than half of the staff actually identified whether or not patients had children. At face value, this seems paradoxical. The workers reported that identification of children was a task that healthcare workers should handle, yet they had not incorporated this task into their own routines. It seems that the accommodating attitudes towards including a child perspective had not been translated into practice.

The reasons why it may be challenging to facilitate change in practice are probably multifactorial (Korhonen et al. 2008b; Maybery and Reupert 2006, 2009). One explanation may be that healthcare workers traditionally focus on problems, rather than focusing on the capacities of parents with a mental illness (Maybery and Reupert 2006). In addition to the problems that patients present, another issue may be that children are not perceived to be the responsibility of adult mental health care (Biebel et al. 2004). As shown in previous research, some of the barriers may be due to organizational factors (Maybery and Reupert 2009). Implementation of new routines is a time consuming effort (Fixsen et al. 2005), and the clinic does not receive additional resources as a consequence of the new legislation. This means that staff members have to include the new routines among the tasks they already are obligated to carry out. Also, the participating clinic in this study covers a vast geographical area, and it may be perceived as difficult to actually access and talk to the children who live in communities far away. This, however, does not clarify or explain the reluctance to identify children of mentally ill patients, and reluctance to talk to patients about their role and responsibilities as parents.

The group that identified children was significantly different from the group that did not in terms of Knowledge Children and Knowledge Legislation. This may indicate that increased knowledge could facilitate the identification of more children (Mayberry et al. 2011). Altered healthcare legislation has changed the terms, and children now formally have the right to be informed and receive adequate follow-up from adult mental health services (Norwegian Ministry of Health and Care Services 2010). There were no differences between the group who identified and the group who did not identify in terms of attitudes. In fact, both groups scored very high on positive attitudes, which may reflect that they respond in a socially desirable way. Another interpretation may be that there was a ceiling effect when measuring attitudes, as the mean scores were almost 4.5 on a five-point scale, which may result in problems differentiating between participants at the top end of the scale. It seems fair to assume that only the worker most interested in the research topic of this study responded. If this is true, a higher response rate in the study could have compensated for such a possible ceiling effect.

In this study, some significant predictors of attitudes were discovered. Those with a medium level of education were more positive towards identifying the children as compared to those with high education. The staff with a medium educational level mainly consists of psychiatric nurses and general nurses. Nurses with additional training in family-focused approaches to treatment, are found to be more open to and active in pursuing a child-focused clinical practice (Korhonen et al. 2008a; 2008b). There is also the possibility that those who have a higher level of education, (i.e., medical doctors and psychologists), were trained in an era when therapists held beliefs that families were a major cause of the problem (Smith and Velleman 2002). Furthermore, focusing on the child and the consequences of parental mental illness for offspring in adult mental health care has only been practiced for the past decade (Aamodt and Aamodt 2005; Korhonen et al. 2008b). Another explanation may be that those with a higher level of education are often those who hold leading positions. Staff with management responsibilities may perceive their position as needing to prioritize the demand for more cost-effective and less time-consuming treatment of patients and may, therefore, not wish to extend the treatment scenario to include the children of patients (Maybery and Reupert 2009; Jones and Scannell 2002).

Furthermore, age was a significant predictor of positive attitudes towards identifying and supporting parents and children in mental health services for adults. One possible explanation for why younger workers are more positive may be that they have a more recent education encompassing a greater focus on the trans-generational transmission of psychopathology and risk factors related to children of mentally ill patients. They may also be more open and flexible than colleagues who have been educated earlier on and been socialized into previously established routines and ways of doing things.

The final predictor of positive attitudes was Expectations Intervention. Perhaps not surprisingly, those who had positive expectations related to changing the routines and the intervention suggested also had more positive attitudes in general towards including a child perspective in the treatment of adults.

We were also able to predict concerns about the patient-therapist relation being disturbed by bringing a family focus into the treatment of mentally ill parents. One possible explanation for a lesser degree of concern from those who report a high knowledge of children and have positive expectations for the new intervention may be that they are more updated on recent research on the benefits of incorporating a family focus in mental health care for adults. This could be due to the recent attention that has been given to the topic in the process of implementing legislative change (Norwegian Health Directorate 2010), as well as further training in family-focused approaches (Korhonen et al. 2008a; 2008b).

### Study Limitations

The study relied solely on self-report measures for attitudes, knowledge, and current work practice. Future studies may also include objective measures, e.g., journal data for assessing the number of children of mentally ill patients. Another limitation was the relatively modest response rate of this study (50 %). However this response rate is similar to mean response rates (49.6 %) in clinical and counseling psychology based on meta-analysis results from 308 surveys (Van Horn et al. 2009). This may have biased the results if the decision to participate was related to worker attitudes, i.e., that those who were already positive about involving the children of their patients were more likely to participate. One consequence may be that this article presents the attitudes within the workforce as being more positive than what they actually are. Although the attitude scale had high reliability, the skewed results in terms of extremely positive attitudes, may be due to the validity of the scale and more specifically how the items were phrased. The scale should be revised to ensure that staff who have truly negative attitudes related to this issue can be measures as such. Future studies should also include other explanatory variables for predicting attitudes, especially for Concerns Patient-Therapist, where only 14 % of the variance was explained. This may include both individual characteristics as well as organizational variables.

Although part of a longitudinal project, the present study is cross-sectional. Even though cross-sectional studies can be useful in assessing practices, attitudes, knowledge and beliefs, they also imply some limitation. In cross-sectional studies it is not possible to say anything about cause and effect. This means that even though knowledge was a significant predictor of attitudes, we have no means of saying it is a lack of knowledge that is causing the low number of children being identified within the participating clinic.

### Conclusion

Changes in legislation or attitudes alone do not necessarily lead to definitive change in practice. Only 44 % of the workers reported identifying children despite reporting very high levels of attitudes towards including a child perspective, and despite the fact that identification is legally required of all mental healthcare workers. We believe the study illustrates the need for implementing a family focus and an awareness of the patient’s parenting role in the education of mental health care workers. Additionally, to improve the knowledge about children and parenting, this should be included as a part of the training in practice. Increasing the level of knowledge may speed up the pace in identifying patient’s children, which lays the foundation for further interventions to prevent mental health problems.
